# Investigation of basic versus additional EAST nudge-based reminders for follow-up examination attendance among workers: a quasi-experimental study

**DOI:** 10.3389/fpubh.2026.1948841

**Published:** 2026-10-14

**Authors:** Masaki Takebayashi, Tatsuya Koyama, Yuri Mizota, Yudai Kaneda, Mira Namba, Hirohide Shibutani, Kouichi Tamura, Yasuo Terauchi, Hirokazu Okada

**Affiliations:** 1 Aomori University of Health and Welfare, Aomori, Japan; 2 Aomori University, Aomori, Japan; 3 Shizuoka Graduate University of Public Health, Shizuoka, Japan; 4 Graduate School of Public Health, Shizuoka Graduate University of Public Health, Shizuoka, Japan; 5 Jyoban Hospital, Iwaki, Japan; 6 Iwaki City Medical Center, Iwaki, Japan; 7 Yokohama City University, Yokohama, Japan; 8 Saitama Medical University, Iruma, Japan

**Keywords:** cognitive bias, employee, nudge, occupational health, secondary examination

## Abstract

**Background:**

Follow-up examinations after abnormal health checkup findings are important for addressing chronic diseases. While Easy–Attractive–Social–Timely (EAST) nudge-based reminders have been used to promote follow-up examination attendance, the appropriate extent of nudge content to include remains unclear. This study investigated the association between basic and additional EAST nudge-based reminders and follow-up examination attendance among workers.

**Methods:**

A quasi-experimental study was conducted among workers at a Japanese transportation company who had abnormal findings and did not respond to an initial notification from October 2024 to March 2025. Participants were geographically assigned to either the basic EAST group (*n* = 210) or the additional EAST group (*n* = 122). The basic EAST group received mailed reminders incorporating one principal design element corresponding to each EAST component: Easy (e.g., specifying the appropriate medical departments), Attractive (e.g., a four-panel comic), Social (e.g., encouraging commitment), and Timely (e.g., “Final” labeling). The additional EAST group received reminders containing the same elements as those provided to the basic EAST group, together with a photograph of an expert, her professional affiliations, and an explanation of the commitment concept (Attractive and Social).

**Results:**

Attendance at follow-up examinations was higher in the basic EAST group than in the additional EAST group (29.0% versus 18.0%; *p* = 0.022; adjusted odds ratio = 1.80; 95% confidence interval, 1.11–3.25).

**Conclusion:**

Although positive outcomes were observed for the basic EAST reminder, the nonrandomized allocation and unequal group sizes warrant cautious interpretation of the findings, and further well-designed studies are needed.

## 1 Introduction

Noncommunicable diseases (NCDs) represent a predominant global public health challenge with particularly severe impacts on the working-age population (1). In 2021, approximately 42% of NCD-related deaths occurred in individuals aged < 70 years, exceeding the combined total number of all other causes of death in this age group (2). The estimated productivity loss attributable to NCDs between 2011 and 2030 is approximately USD 30 trillion, underscoring the need for urgent action (3). The World Health Organization emphasizes equitable access to early detection and timely treatment as key components of NCD management and suggests that early intervention for NCDs yields a high return on investment (4, 5).

In Japan, regular health checkups are mandatory for workers, and equitable access is guaranteed institutionally (6). However, follow-up examinations for individuals with abnormal findings are not mandatory, and the follow-up rate for individuals with hypertension, diabetes, and dyslipidemia remains at approximately 30% (7). Initial and reminder notifications are widely used to promote follow-up examination attendance in occupational health settings. Although reminder notifications have the potential to improve attendance among individuals who do not respond to the initial notification, some workers still fail to undergo the recommended follow-up examinations (8–10). Previous studies have suggested that individuals who do not undergo these examinations may exhibit present bias (11, 12). A follow-up examination represents a typical intertemporal choice (in which current efforts yield future benefits), and individuals with a stronger present bias may procrastinate in attending even when provided with information regarding health risks (12). Therefore, to increase attendance rates, it is crucial to design notifications that account for such cognitive biases.

Nudging has been proposed as an effective intervention for individuals who fail to engage in healthy behaviors because of cognitive biases (13). It is defined as “any aspect of the choice architecture that alters people’s behavior in a predictable manner without forbidding any options or significantly changing their economic incentives” (13). A representative nudge framework is the Easy–Attractive–Social–Timely (EAST), introduced in an official report by the Behavioural Insights Team, which was originally established within the United Kingdom government (14). For example, reducing barriers to action (Easy nudge) may encourage individuals with present bias to initiate healthy behaviors (14). Nudges are generally more cost-effective than other interventions and may be particularly well suited to occupational health settings with limited personnel or budgets (15). The Japanese government recommends the use of nudges in the workplace and promotes the EAST framework to increase cancer screening uptake (16, 17). Among the good practices presented in a Japanese government report, the largest improvement in cancer screening uptake was a 17-percentage-point increase from 36 to 53%, following the implementation of an Easy nudge (17). A previous report aimed at providing a practical framework for applying behavioral science to public policy suggested that the most effective interventions will combine different elements (18). However, incorporating too many components may increase cognitive friction, thereby weakening the effectiveness of the intervention (14). Because nudging encompasses a wide range of strategies, occupational health practitioners often face challenges in selecting appropriate intervention components (19). Examining whether reminder notifications should rely solely on the EAST framework or incorporate additional nudge elements offers valuable insights for occupational health practices, as no field studies have yet addressed this issue in this context.

This study aimed to investigate the association between basic and additional EAST-based reminder notifications and follow-up examination attendance among workers.

## 2 Methods

### 2.1 Research design

A quasi-experimental study with two parallel groups was conducted, and the results were reported in accordance with the Transparent Reporting of Evaluations with Nonrandomized Designs (TREND) statement (20).

### 2.2 Setting

The study was conducted at a Japanese transportation company with 100 centers nationwide and a standardized company-wide occupational health management system, including annual health checkups.

### 2.3 Study population

The study population consisted of workers who had abnormal health checkup findings and received a reminder notification for follow-up examinations during the latter half of fiscal year 2024 (October 2024 to March 2025).

### 2.4 Eligibility criteria

No separate recruitment procedure was conducted; eligible workers were identified through the company’s routine occupational health process. The inclusion criterion was workers who did not respond to the initial notification within three weeks. The exclusion criteria included workers who took leave or resigned during the study period.

### 2.5 Group allocation and intervention

Participants were allocated to either the basic EAST or additional EAST group based on their worksite location: 54 centers in eastern and central Japan were assigned to the basic EAST group, and 46 centers in western Japan were assigned to the additional EAST group. This geographical allocation was chosen because of its operational feasibility and to minimize the cross-contamination of intervention materials within the same regional cluster.

Workers with abnormal findings on their routine health checkups initially received a mailed notification from the company encouraging them to undergo follow-up examinations. The initial notification incorporated an Easy nudge by explicitly indicating the appropriate medical department for follow-up examinations (21) (Figure 1A). Recipients were instructed to report their appointment status. If they did not respond within three weeks, a reminder notification was mailed. In occupational health practice, reminder notifications are sometimes similar in content to the initial notifications; however, such repetition can induce decision inertia, leading recipients to disregard the message (22). Therefore, in this study, reminder notifications were deliberately differentiated from the initial notification. The notification design was reviewed by two external experts in behavioral economics for consistency with the EAST framework. In addition, the reminder notifications were pilot-tested with 20 workers (10 men and 10 women) from other companies who had abnormal health checkup findings, and their verbal feedback was incorporated into the final design. Both the initial and reminder notifications were printed in black and white on A4-sized paper.

**Figure 1:**
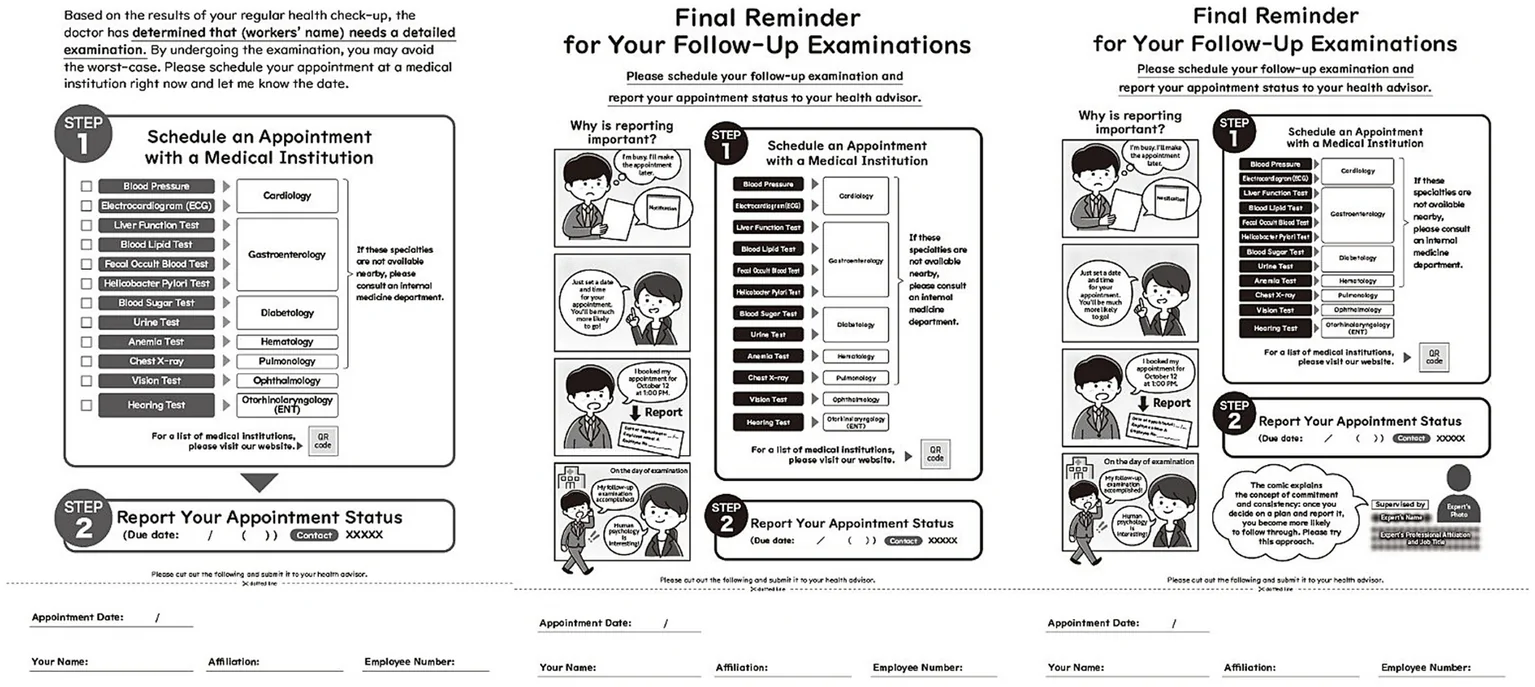
Notifications for follow-up examinations. **(A)** (left): Initial notification for both groups. **(B)** (middle): Reminder notification for the basic EAST group (partially modified by some centers). **(C)** (right): Reminder notification for the additional EAST group. For privacy reasons, the expert’s photograph, name, professional affiliations, and job titles were masked.

#### 2.5.1 Basic EAST group

The basic EAST reminder incorporated one principal design element corresponding to each EAST component (Figure 1B). While retaining the Easy nudge incorporated in the initial notification, the following elements implemented the remaining components of the EAST framework: a four-panel comic designed to attract attention (Attractive nudge), encouraging participants to report their appointment status as a commitment (Social nudge), and the word “Final” to the title to emphasize the approaching deadline (Timely nudge) (14).

#### 2.5.2 Additional EAST group

In addition to the nudge elements used in the basic EAST group, the notification for this group included a photograph of an expert, along with her name, professional affiliations, and job titles, as well as an additional explanation of the commitment concept (Attractive and Social nudges) (23–25). She was selected based on her expertise in behavioral science and her involvement in developing national cancer screening strategies (Figure 1C). She was one of the authors and served as a supervising expert for a Japanese government handbook on promoting cancer screening uptake (17). She had no employment or consulting relationship with the participating company.

### 2.6 Outcomes

The primary outcome was attendance at follow-up examinations by June 30, 2025. Completion of the follow-up examinations was assessed based on workers’ self-reports and subsequently verified using medical claims data submitted by healthcare providers. The secondary outcome was the reported appointment status.

### 2.7 Statistical analysis

Following a previous study, a sample size of 198 participants (99 in each group) was computed using G*Power (version 3.1.9.4; Heinrich Heine University, Düsseldorf, Germany), assuming 80% power, an α error of 5%, and an effect size of 20% (26). Categorical variables were analyzed using Pearson’s χ^2^ test, and continuous variables were analyzed using t-tests. Unadjusted odds ratios (ORs) and 95% confidence intervals (CIs) were estimated using univariable logistic regression. Adjusted ORs and 95% CIs were estimated using mixed-effects logistic regression, with sex, age, and the number of abnormal findings included as covariates and the center included as a random effect to account for clustering at the center level. *p* values for group differences were obtained using the Wald χ^2^ test within the same mixed-effects logistic regression model. Data were analyzed using SPSS software (version 28; IBM, Tokyo, Japan). Statistical significance was set at *p* < 0.05 (two-tailed).

### 2.8 Ethical considerations

The study protocol was approved by the Research Ethics Committee of Aomori University (Approval No. 06-2023) and registered with the University Hospital Medical Information Network Clinical Trials Registry (UMIN000057239). All workers were informed via the company intranet that their data would be used for research purposes only and that participation was voluntary under an opt-out approach. Informed consent was obtained in writing from the company’s management. After completing the study, participants were given the opportunity to review both notification types. Adverse events, including participant complaints, were actively monitored throughout the study period. Dedicated contact points for reporting complaints or adverse events were established both at the research ethics committee of the first author’s institution and within the occupational health division of the participating company. In addition, designated occupational health staff at each worksite routinely monitored and recorded any inquiries, complaints, or other feedback from workers. Some occupational health practitioners may have ethical concerns regarding autonomy and manipulation of nudges, including the perception that nudging is covert and that transparent disclosure may undermine its effectiveness (27). To address these concerns, all workers were informed in advance of the use of nudges via the company’s intranet. Moreover, the use and underlying mechanism of the commitment nudge were transparently disclosed through a four-panel comic in the reminder notifications.

## 3 Results

A total of 7,280 workers (6,495 men and 785 women; mean age 49.7 years) underwent routine health checkups, with 4,574 in the basic EAST group and 2,706 in the additional EAST group. Of these, 961 had abnormal findings requiring follow-up examinations: 610 in the basic EAST group (576 men and 34 women; mean age 50.5 years) and 351 in the additional EAST group (332 men and 19 women; mean age 50.5 years). After excluding those who responded to the initial notification and those who resigned, 210 workers in the basic EAST group and 122 in the additional EAST group were included in the analysis (Figure 2). The response rates to the initial notification were similar between the two groups: 62.1% (379/610) in the basic EAST group and 61.8% (217/351) in the additional EAST group (*p* = 0.925).

**Figure 2:**
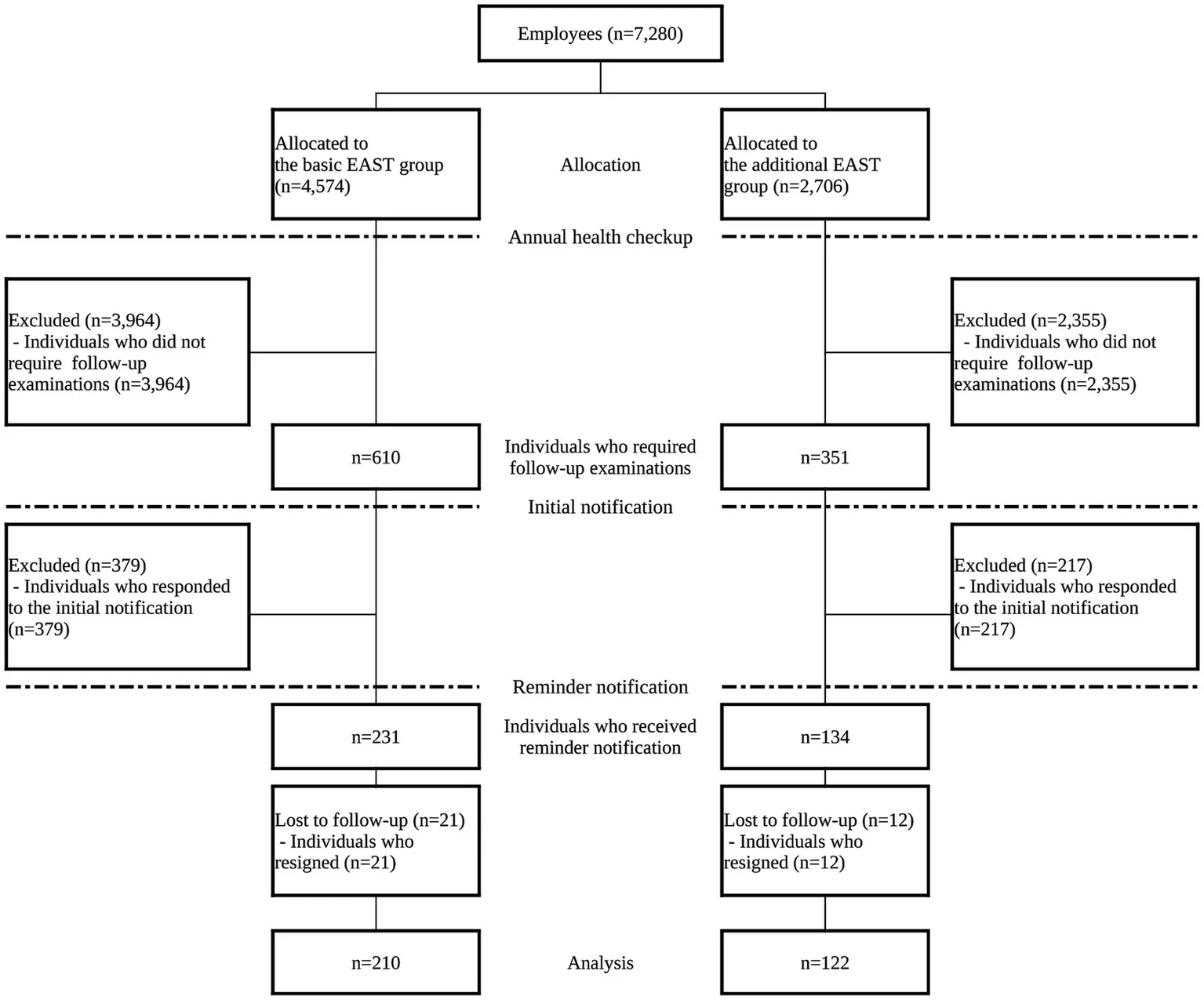
Participant flow diagram.

No significant differences were observed in the baseline characteristics between the groups (Table 1). Workers in the basic EAST group were more likely to report their appointment status than those in the additional EAST group (*p* = 0.026; unadjusted OR = 2.05; 95% CI, 1.07–3.91; adjusted OR = 2.04; 95% CI, 1.08–3.83). Similarly, workers in the basic EAST group were more likely to complete follow-up examinations (*p* = 0.022; unadjusted OR = 1.88; 95% CI, 1.07–3.23; adjusted OR = 1.80; 95% CI, 1.11–3.25) (Table 2).

**Table 1:** Basic attributes of participants.

	Basic EAST group (*n* = 210)		Additional EAST group (*n* = 122)		*p* value
	*n*, mean	%, ±SD	*n*, mean	%, ±SD	
Sex					0.807
Male	197	93.8%	116	95.1%	
Female	13	6.2%	6	4.9%	
Age, years	49.5	±10.7	49.0	±10.5	0.684
Number of abnormal findings	1.2	± 0.6	1.1	± 0.6	0.144

Pearson’s χ^2^ test was conducted for sex; a t-test was conducted for age and number of abnormal findings.

SD, standard deviation.

**Table 2:** Outcomes between the basic and additional EAST groups.

	Basic EAST group (*n* = 210)	Additional EAST group (*n* = 122)	*p* value^a^	Unadjusted OR (95% CI)^b^	Adjusted OR (95% CI)^c^
Workers who reported appointment status	44 (21.0%)	14 (11.5%)	0.026	2.05 (1.07–3.91)	2.04 (1.08–3.83)
Workers who attended follow-up examinations	61 (29.0%)	22 (18.0%)	0.022	1.88 (1.07–3.23)	1.80 (1.11–3.25)

OR, odds ratio; CI, confidence interval.

ᵃp values for group differences were obtained using the Wald χ^2^ test within the mixed-effects logistic regression model.

^b^Unadjusted ORs and 95% CIs were estimated using univariable logistic regression.

^c^Adjusted ORs and 95% CIs were estimated using mixed-effects logistic regression, with sex, age, and the number of abnormal findings included as covariates and center included as a random effect to account for clustering at the center level.

No adverse events were reported during the study period.

## 4 Discussion

### 4.1 Overview

Although geographic allocation resulted in unequal group sizes, with a 1:1.7 ratio between the additional and basic EAST groups (122 versus 210 participants), baseline characteristics and response rates to the initial notification were comparable between the two groups. Both appointment-status reporting and follow-up examination attendance were significantly higher in the basic EAST group than those in the additional EAST group. The unadjusted and adjusted ORs were similar for both outcomes, suggesting that adjustment for the covariates included in the model may have had little influence on the estimated associations. The potential explanations for these results are discussed below.

These findings suggest that the basic EAST reminder, which incorporated one principal design element corresponding to each EAST component, may represent a practical approach in the present context. Because the components of the basic EAST reminder were not evaluated separately, their individual contributions could not be determined. Nevertheless, several features of the reminder may help interpret the association. The Easy nudge, which simplifies information and facilitates decision-making, has been reported to have a larger effect size than other nudges, suggesting that preserving simplicity may be important, even when combining multiple nudge elements (28). Regarding the Attractive and Social nudge elements, the four-panel comic explaining the concept of commitment may have attracted attention and increased engagement with the message. This interpretation is consistent with previous studies suggesting that comics and theory-based explanations may facilitate understanding and engagement with behavioral messages (29, 30). The Timely nudge, which explicitly labeled the notification as “Final,” may have helped increase the salience of the deadline and reduce procrastination (31).

Meanwhile, the additional EAST group had significantly lower outcomes. We did not expect such a marked difference because the additional components were based on evidence that messages endorsed by authority figures and accompanied by a person’s photograph may be more persuasive (23–25). One possible explanation for this difference is that the additional information may have reduced the simplicity of the reminder. The repeated explanation of commitment may have evoked message fatigue (i.e., tiredness caused by repeated exposure to similar messages) (32). Furthermore, the additional components resulted in a denser layout and smaller font sizes. Given the participants’ mean age of approximately 50 years, these design changes may have reduced readability and cognitive ease (a psychological state in which easily processed information tends to be perceived as more trustworthy), potentially compromising receptiveness to the reminder (33, 34). This interpretation is consistent with previous research suggesting that even minor negative emotions and increased information can interfere with desirable decision-making (14, 18, 32–35). The present findings suggest that maintaining the simplicity of the core EAST components may be preferable to adding supplementary elements that increase information burden, particularly given the potential for additive bias (a tendency to favor addition) when designing interventions (36).

When interpreting these findings, sex differences and the characteristics of the transportation industry warrant consideration. Women have been reported to be less likely than men to attend follow-up health guidance in Japan (37). The participating company had a predominantly male workforce, consistent with the wider Japanese transportation sector (38). Therefore, the present findings may be particularly relevant to transportation companies with similar workforce characteristics.

### 4.2 Strengths

This study had two strengths from the perspective of occupational health practice. The first was the potentially lower implementation cost of the basic EAST reminder, which may be better aligned with the practical needs of employers and occupational health practitioners. Specifically, compared with the additional EAST reminder, the basic EAST reminder required minor modifications to the initial notification format, and omitting expert information may reduce both preparation effort and printing costs. Second, no adverse events were reported, even though the nudges used in the notifications were transparently disclosed. Given that ethical concerns about nudges are widespread and that this study was conducted in Japan, which has been classified as the most conservative category with respect to attitudes toward nudging, these findings may provide preliminary reassurance to occupational health practitioners considering the use of transparent nudges (27, 39).

### 4.3 Limitations

This study has several limitations that should be considered when interpreting the findings. First, owing to operational constraints, a randomized controlled trial could not be conducted. Despite adjusting for age, sex, and the number of abnormal findings and accounting for clustering at the center level, residual confounding factors (e.g., regional cultural differences in communication preferences) due to nonrandom allocation could not be ruled out. Due to the limited number of female participants, we were unable to obtain sufficient data to evaluate outcomes specifically among women. Moreover, geographic allocation resulted in unequal group sizes, which may have affected the precision of the estimates. Therefore, causal inference was limited. Second, contamination between groups may have occurred because the study was conducted within a single company, and information may have been shared among workers. Third, the mechanisms proposed to explain the findings, including information overload, message fatigue, and reduced cognitive ease, were not directly assessed in this study but were instead inferred from previous studies (14, 18, 32–35). Fourth, it was not possible to determine whether any specific component contributed to the lower attendance observed in the additional EAST group because multiple elements were added simultaneously. Future qualitative studies are needed to explore perceptions of reminder designs, identify barriers to follow-up examination attendance, and elucidate sex differences in responses to the message and messenger. Fifth, even after the basic EAST reminder was sent, only 29.0% of the recipients attended the follow-up examinations. A comparison with other nudge intervention groups would have been valuable. However, owing to operational constraints, such comparisons were not feasible in the present study. Finally, this study was conducted at a single company, which limits the generalizability of the findings to other settings. Further studies, including large-scale randomized controlled trials, are required to address these limitations.

## 5 Conclusion

The results suggest that the basic EAST reminder may be a practical option in settings with limited resources. However, given the nonrandomized allocation and unequal group sizes, further well-designed studies are needed to confirm these findings.

## Data Availability

Due to confidentiality restrictions, the datasets analyzed in the current study are not publicly available. These datasets are available from the corresponding author upon request.
